# The cellular response to ocean warming in *Emiliania huxleyi*

**DOI:** 10.3389/fmicb.2023.1177349

**Published:** 2023-05-15

**Authors:** Craig J. Dedman, Samuel Barton, Marjorie Fournier, Rosalind E. M. Rickaby

**Affiliations:** ^1^Department of Earth Sciences, University of Oxford, Oxford, United Kingdom; ^2^Advanced Proteomics Facility, Department of Biochemistry, University of Oxford, Oxford, United Kingdom

**Keywords:** ocean warming, proteomics, environmental change, phytoplankton, marine biology

## Abstract

Marine phytoplankton contribute substantially to the global flux of carbon from the atmosphere to the deep ocean. Sea surface temperatures will inevitably increase in line with global climate change, altering the performance of marine phytoplankton. Differing sensitivities of photosynthesis and respiration to temperature, will likely shift the strength of the future oceanic carbon sink. To further clarify the molecular mechanisms driving these alterations in phytoplankton function, shotgun proteomic analysis was carried out on the globally-occurring coccolithophore *Emiliania huxleyi* exposed to moderate- (23°C) and elevated- (28°C) warming. Compared to the control (17°C), growth of *E. huxleyi* increased under elevated temperatures, with higher rates recorded under moderate- relative to elevated- warming. Proteomic analysis revealed a significant modification of the *E. huxleyi* cellular proteome as temperatures increased: at lower temperature, ribosomal proteins and photosynthetic machinery appeared abundant, as rates of protein translation and photosynthetic performance are restricted by low temperatures. As temperatures increased, evidence of heat stress was observed in the photosystem, characterized by a relative down-regulation of the Photosystem II oxygen evolving complex and ATP synthase. Acclimation to elevated warming (28°C) revealed a substantial alteration to carbon metabolism. Here, *E. huxleyi* made use of the glyoxylate cycle and succinate metabolism to optimize carbon use, maintain growth and maximize ATP production in heat-damaged mitochondria, enabling cultures to maintain growth at levels significantly higher than those recorded in the control (17°C). Based on the metabolic changes observed, we can predict that warming may benefit photosynthetic carbon fixation by *E. huxleyi* in the sub-optimal to optimal thermal range. Past the thermal optima, increasing rates of respiration and costs of repair will likely constrain growth, causing a possible decline in the contribution of this species to the oceanic carbon sink depending on the evolvability of these temperature thresholds.

## 1. Introduction

Ocean warming appears the most imminent threat to the marine ecosystem as a result of global climate change. During the next century, sea surface temperatures are estimated to increase 1°C–6°C and display greater fluctuation due to extreme weather events (IPCC, 2007, 2013; Oliver et al., 2021). Marine phytoplankton drive the oceanic carbon cycle, acting as the base of the marine food chain and contributing ~50% of global primary productivity (Field et al., 1998). Through photosynthesis, marine phytoplankton fix carbon from the atmosphere, utilizing the products of photosynthesis to fuel respiration, and growth (Raven and Geider, 1988). In this manner, phytoplankton play a key role in mitigating global climate change as the primary vector for the “biological carbon pump,” whereby fixed carbon is subsequently transported via sinking from the surface waters to the deep ocean, representing a major global carbon sink (Passow and Carlson, 2012; Le Quéré et al., 2016; Boscolo-Galazzo et al., 2018). The direct impact of temperature upon phytoplankton growth has proved a subject of interest for a number of years (Eppley, 1972; Raven and Geider, 1988; Thomas et al., 2012; Anderson et al., 2021), and holds particular importance for assessing the oceanic carbon sink in the face of ocean warming. Nonetheless, our understanding of its impact on phytoplankton physiology is somewhat incomplete (Toseland et al., 2013), particularly in regard to the molecular mechanisms which drive phytoplankton’s response to warming. Gaining a comprehensive understanding of the impacts of ocean warming on phytoplankton function is critical to evaluating the likely effect on the wider marine ecosystem and global biogeochemical cycles (Falkowski et al., 1998; Field et al., 1998; Falkowski, 2012; Laws, 2013).

Temperature increases the rate of many metabolic reactions because of enhanced enzymatic activity (Somero, 1995), affecting a number of physiological processes including translation, respiration, and photosynthetic carbon fixation (Falkowski and Raven, 1997; Brown et al., 2004; Allen et al., 2005; Xu et al., 2011; Toseland et al., 2013; Barton et al., 2020). The relationship between temperature and phytoplankton performance has typically been assessed using thermal reaction norms, or tolerance curves (Boyd et al., 2013; Baker et al., 2016; Bestion et al., 2018; Barton and Yvon-Durocher, 2019). Generally, phytoplankton growth and metabolism (i.e., photosynthesis and respiration) follow a negatively skewed relationship with temperature, whereby rates increase until they reach an optimum, after which subsequent rises cause a rapid decline (Schoolfield et al., 1981; Kingsolver, 2009; Thomas et al., 2012; Barton and Yvon-Durocher, 2019). Net fixation of carbon by marine phytoplankton is largely determined by the balance between photosynthesis and respiration (Field et al., 1998). At both the species- and community- scale, research has shown that these two processes display different sensitivities toward temperature (Brown et al., 2004; Allen et al., 2005). In experiments on a range of marine phytoplankton mono-cultures, respiration consistently displayed a higher thermal sensitivity and optimum temperature than photosynthesis during short-term exposure, compromising carbon fixation (Barton et al., 2020). When considered on a community scale, ocean warming will likely disrupt the metabolic balance of the ocean by increasing the rate of respiration relative to primary production (Laws et al., 2000; Lopez-Urrutia et al., 2006; Regaudie-De-Gioux and Duarte, 2012; Boscolo-Galazzo et al., 2018), causing a reduction in the contribution of phytoplankton to the oceanic carbon sink. This effect on the carbon sink, however, will depend on the ability of the phototrophic community to adapt their metabolism to warming temperatures, which will likely vary by species, region and rate of warming.

Advances in omics technologies have broadened our ability to consider the impact of environmental perturbations upon biota. A number of transcriptome studies have been carried out to examine the molecular pathways most impacted by temperature in marine phytoplankton at the gene expression level, often in conjunction with an additional environmental stressors such as ocean acidification (Thangaraj and Sun, 2021; Jin et al., 2022; Liang et al., 2023; Thangaraj et al., 2023; Zhang et al., 2023). Proteomic analysis, the examination of organismal response at the individual protein scale, can enhance our understanding of the metabolic response. It is likely that the response to temperature may exist within a complex network of metabolic and biochemical processes (O’Donnell et al., 2018). Hence, proteomic analysis may provide additional mechanistic insight toward temperature-dependent functions such as growth, photosynthesis and respiration. To date, limited evidence is available on the proteomic response of phytoplankton toward ocean warming, restricted to a few taxa (Thangaraj et al., 2020a,b; Cheng et al., 2022).

In this study we employed the ubiquitous coccolithophore, *Emiliania huxleyi*, a globally occurring phytoplankton species of high ecological importance (Paasche, 2001; Jones et al., 2011), as a model to examine the cellular response of phytoplankton toward ocean warming. Cultures were exposed to moderate- and elevated- warming conditions, which, respectively, appeared to represent conditions in the sub- and supra-optimal thermal range for this strain, before shotgun proteomic analysis was used to examine any alteration in cellular function. Analysis revealed a significant alteration in abundance of photosynthetic and respiratory proteins, in-line with earlier work, providing additional insight on the mechanisms driving their contrasting sensitivity to temperature. A reduction in photosynthetic investment due to its enhanced efficiency with temperature, coupled with improved translation efficiency, appears likely to benefit growth in the sub-optimal to optimal temperature range, enhancing the incorporation of carbon into organic biomass. Additionally, we identified an ability of *E. huxleyi* to modify its carbon metabolism to sustain growth at elevated temperature when experiencing mitochondrial damage. Our work suggests that increases in ocean temperature in the sub-optimal range of thermal tolerance may enhance carbon fixation by *E. huxleyi*. However, at temperatures beyond the thermal optima of growth, and in the absence of an evolutionary response, increasing costs of repair and reduced photosynthetic energy are likely to constrain growth and possibly the contribution of this species to the oceanic carbon sink.

## 2. Methods

### 2.1. Experimental conditions

*Emiliania huxleyi* strain RCC911 was obtained from the Roscoff Culture Collection and routinely maintained in K/2 media at 17°C, in-line with its ancestral culturing conditions. This strain was originally isolated from the Pacific Ocean at a temperature of 28°C, but has been routinely maintained at 17°C since its isolation in 2004 and therefore can be considered to be adapted to this temperature. To establish cultures for warming experiments, a dilute inoculum was added to Aquil media (Morel et al., 1979) held within filter-capped tissue culture flasks (50–200 mL) under sterile conditions. Growth media was filter-sterilized before use (0.22 μm) to avoid bacterial contamination. Flasks were placed within individual PHCbi MLR-352-PE Incubators (PHC Europe B.V.) set at one of three temperatures; 17°C, representing the recommended incubation temperature for this strain and therefore utilized as a control; and two warming treatments representing, (i) moderate warming (23°C) and (ii) elevated warming (28°C), respectively. Triplicate cultures were prepared for all treatments, representing three biological replicates. Lighting conditions were identical across each treatment (30–50 μmol m^−2^ s^−1^), under a 14:10 h (light:dark) regime. Cultures were exposed to experimental conditions by weekly transfers into fresh culture media during the exponential growth phase for 6 weeks representing ~40 generations. When inoculating fresh media, efforts were made to add an identical inoculum cell density between treatments, roughly 10^3–4^ cells mL^−1^. Cell density of *E. huxleyi* cultures grown at each temperature were routinely monitored using a Beckman Z2 Coulter Counter. Briefly, a 1 mL sub-sample was collected from culture flasks and diluted 10× in 3.5% NaCl prior to analysis. Cell counts were converted to cells mL^−1^ and the specific growth rate calculated using the formula ln(*n*2/*n*1)/(*t*2−*t*1).

### 2.2. Shotgun proteomic analysis

#### 2.2.1. Harvesting of cellular material for proteomic analysis

*Emiliania huxleyi* cultures belonging to each treatment were harvested during mid-late exponential growth of the final sixth transfer. Here, 150 mL of culture was collected by light centrifugation at 2,500 rpm for 10 min at 4°C in order to prevent physical damage to cells and hence leakage of cellular content into the supernatant. Subsequently, the supernatant was discarded and cells to be processed for shotgun proteomic analysis were washed 3x via centrifugation (2,500 rpm for 10 min at 4°C) in SOW. Cell pellets were immediately flash-frozen using dry ice and stored at-20°C until further processing.

#### 2.2.2. Protein extraction and digestion

To prepare cell pellets for shotgun proteomic analysis, cell pellets were thawed at room temperature and subsequently lysed by sonication (6 × 30 s, on ice) within 1 mL extraction buffer (20 mM Tris–HCl, pH 8.0), as previous (Zhang et al., 2018). Following this, a Bradford assay (Bradford, 1976) was used to determine the concentration of protein present in each sample. The resultant cell lysate was transferred to a fresh Eppendorf tube and stored at-20°C. In-solution trypsin digestion was carried out to digest 100 μg of protein from each sample. Briefly, proteins were denatured in 4 M urea in 100 mM ammonium bicarbonate for 10 min at room temperature under shaking (650 rpm). Following this, cysteines were reduced using 10 mM tris(2-carboxyethyl)phosphine (TCEP) for 30 min at room temperature, and subsequently alkylated with 50 mM iodoacetamide for a further 30 min in the dark. Proteins were pre-digested using LysC at a ratio of 1 μg per 100 μg protein, and incubated for 2 h at 37°C under shaking (800 rpm). Urea concentrations were diluted to 2 M and CaCl_2_ added at a final concentration of 2 mM, before trypsin was added at a ratio of 2.5 μg for every 100 μg protein. The trypsin reaction was carried out for 20 h (37°C, 800 rpm) and stopped by the addition of 5% formic acid. Samples were subsequently desalted using a C18 column and dried overnight using a speed-vacuum, and stored at-20°C until analysis.

#### 2.2.3. Mass spectrometry

Peptides were separated using nano liquid chromatography (Thermo Fisher Scientific Ultimate RSLC 3000) coupled with a Q Exactive mass spectrometer equipped with an Easy-Spray source (Thermo Fisher Scientific). After separation, peptides were trapped onto a C18 PepMac100 precolumn (300 μm i.d.x5 mm, 100 Å, Thermo Fisher Scientific) using 0.1% formic acid diluted in HPLC grade water. Peptides was further separated using an Easy-Spray RSLC C18 column (75um i.d., 50 cm length, Thermo Fisher Scientific) during a 60-min linear gradient of 0.1% formic acid in acetonitrile (15–35%) at a flow rate of 200 nl min^−1^. Raw data was acquired in data-dependent acquisition mode (DDA). Full scan mass spectra were acquired in the Orbitrap (Scan range 350–1,500 m/z, resolution 70,000; AGC target, 3^6^, maximum injection time, 50 ms). The 10 most intense peaks were selected for higher-energy collision dissociation (HCD) fragmentation at 30% of normalized collision energy. HCD spectra were acquired in the Orbitrap at resolution 17,500, AGC target 5^4^, maximum injection time 120 ms with fixed mass at 180 m/z. Charge exclusion was selected for unassigned and 1+ ions. The dynamic exclusion was set to 20 s.

#### 2.2.4. Analysis

MaxQuant version 2.0.3.0 was used to identify peptides from raw spectral files and carry out label-free quantification (LFQ) of proteins (Cox and Mann, 2008; Cox et al., 2014). Here, the MaxLFQ workflow was used with default settings to generate normalized LFQ intensities, representing the relative protein abundance across all proteins identified during analysis (Cox et al., 2014). LFQ values were only generated for proteins where a minimum of two peptides were present in each sample. To act as a reference for peptide identification the Uniprot reference proteome for *E. huxleyi* (strain CCMP1516; UP000013827) was downloaded on the 21/02/22 (UniProt, 2021). Perseus version 1.6.15.0 was utilized for downstream data analysis (Tyanova et al., 2016). Data was filtered to remove potential contaminants and identified proteins were filtered to include only those present in three replicates of at least one treatment. The mass spectrometry proteomics data have been deposited to the ProteomeXchange Consortium via the PRIDE partner repository with the dataset identifier PXD038243 (Perez-Riverol et al., 2022).

### 2.3. Statistical analysis

ANOVA and post-hoc T-tests (*p* ≤ 0.05) were carried out to identify significant differences in growth of *E. huxleyi* between cultures grown at each temperature. Pair-wise T-tests (FDR corrected q-value ≤0.01) were utilized to examine significant differences in abundance of individual proteins based on LFQ intensity between each treatment. Lists of significantly up- or down- regulated proteins identified in each pair-wise T-test were submitted to DAVID functional enrichment analysis using default settings (Bonferroni FDR ≤ 0.05), such analysis has previously been utilized to examine alterations in the proteome of *E. huxleyi* in response to trace nutrient limitation (Huang et al., 2009; Shire and Kustka, 2021; Sherman et al., 2022).

## 3. Results and discussion

### 3.1. Growth of *Emiliania huxleyi* at varying temperature

To investigate the impact of ocean warming on cell function, the coccolithophore *E. huxleyi* strain RCC911 was grown at 17°C (control) and two warming conditions; moderate- (23°C) and elevated- (28°C) for a period of 6 weeks. Upon harvesting of cultures for shotgun proteomic analysis, a significant increase in the specific growth rate (μ) of *E. huxleyi* was recorded in warming treatments compared to the control (Table 1). No significant difference in μ was recorded between cultures grown at either warming treatment, although μ was on average lower under elevated- (28°C) compared to moderate- (23°C) warming. Considering the negatively-skewed relationship between phytoplankton growth and temperature, and previous research on the thermal tolerance of *E. huxleyi* (Barton and Yvon-Durocher, 2019), it appears that the thermal optima for growth of *E. huxleyi* RCC911 lies between 23°C and 28°C, below its isolation temperature. Of course, the temperature at which a strain is isolated represents a snap-shot in time whereby surface water temperatures may have been particularly warm, or this strain may exist at supra-optimal temperatures close to its upper limits of tolerance which is sometimes the case (Thomas et al., 2012). This finding is largely in-line with previous examination of the optimal temperature for *E. huxleyi*, which has been described for a number of strains in the range of 20–27°C (Van Rijssel and Gieskes, 2002; Sorrosa et al., 2005; Langer et al., 2009; De Bodt et al., 2010; Fielding, 2013; Rosas-Navarro et al., 2016; Barton and Yvon-Durocher, 2019). Hence, the warming treatments examined appear to represent sub- (23°C) and supra-optimal (28°C) warming conditions for this strain. Generally, evidence suggests that growth of *E. huxleyi* is expected to increase with temperature within the range of 2–27°C (Fielding, 2013), however strain-specific differences dependent on the natural environment in which they are isolated are expected. The individual responses of different *E. huxleyi* strains, as well as other phytoplankton, to changing sea surface temperatures will likely influence their geographical distribution in the future ocean and input toward major biogeochemical cycles (Winter et al., 2014; Krumhardt et al., 2017).

**Table 1:** Specific growth rate (*μ*) of *Emiliania huxleyi* cultures harvested for proteomic analysis.

Temperature	*μ*
17°C	0.81 ± 0.01
23°C	1.14 ± 0.04*
28°C	1.02 ± 0.06*

* denotes significance versus 17°C control [two-way T-test (*p* ≤ 0.05)].

### 3.2. Proteomic analysis

#### 3.2.1. Overview

Shotgun proteomic analysis of *E. huxleyi* RCC911, revealed a significant reorganization of cellular function in response to rising temperature. Of high ecological significance, in-line with previous research our results displayed a reprogramming of photosynthesis and respiration, the two primary carbon fluxes of the ocean biological pump (Field et al., 1998; Padfield et al., 2016; Barton et al., 2020). After filtering of data to remove potential contaminants and proteins which were not present in all three replicates of at least one treatment, a total of 1,367 proteins were utilized for downstream analysis. Of these proteins, 586 were present in all replicates of every treatment. ANOVA analysis revealed that 473 of these proteins statistically varied in abundance between treatments (*q* ≤ 0.01), highlighting the considerable impact of varying temperature on the cellular proteome of *E. huxleyi*. Subsequent pair-wise T-tests were utilized to examine significant alterations in the Log2 fold-change (FC) of proteins present in each warming treatment, with respect to the 17°C control, as well as between the two independent warming treatments (*q* ≤ 0.01). A full list of proteins identified is available in Supplementary Table 1.

Enrichment analysis revealed key features (e.g., Gene Ontology Terms; KEGG pathways; InterPro annotation), driving the significant variations observed under warming and facilitated the identification of protein groups for further analysis (Table 2; Supplementary Table 1). Notably, similar features were enriched in both the moderate- and elevated- warming treatments, suggesting their relation to increasing temperature. Briefly, the most enriched terms were derived from down-regulated proteins relative to the 17°C control, identifying translation- and chloroplast- related terms to be key features of the warming response. Similarly, terms related to chlorophyll biosynthesis, porphyrin metabolism and ATP synthase were also enriched in down-regulated proteins relative to the 17°C control (*p* ≤ 0.05). Up-regulated features were also shared between both warming treatments, namely those related to the mitochondria, protein folding and heat shock protein/chaperone activity (*p* ≤ 0.05). In the following sections, the processes highlighted by enrichment analysis to be most affected by warming will be explored in greater detail.

**Table 2:** Significantly enriched features of up- and down- regulated proteins under warming.

Score	Significant features*	*p*-value
*Moderate warming (23°C vs. 17°C)*		
Up-regulated proteins		
1.84	INTERPRO—IPR019809:Histone H4, conserved site	<0.001
	Uniprot—DOMAIN:CENP-T_C	<0.001
	INTERPRO—IPR001951:Histone H4	<0.001
1.48	KEGG Pathway—ehx00280:Valine, leucine and isoleucine degradation	0.006
1.46	PIR_SUPERFAMILY—PIRSF002583:heat shock protein, HSP90/HTPG types	0.004
	INTERPRO—IPR020568:Ribosomal protein S5 domain 2-type fold	0.004
	INTERPRO—IPR001404:Heat shock protein Hsp90	0.009
1.43	GO Term—GO:0005743 ~ mitochondrial inner membrane	0.011
	INTERPRO—IPR001107:Band 7 protein	0.016
	Uniprot—DOMAIN:PHB	0.018
1.16	GO Term—GO:0005524 ~ ATP binding	0.045
Down-regulated proteins		
4.53	GO Term—GO:0003735 ~ structural constituent of ribosome	<0.001
	GO Term—GO:0006412 ~ translation	<0.001
	Uniprot—KW-0689 ~ Ribosomal protein	<0.001
3.92	Uniprot—KW-0150 ~ Chloroplast	<0.001
	GO Term—GO:0009536 ~ plastid	<0.001
	Uniprot—KW-0934 ~ Plastid	<0.001
2.33	GO Term—GO:0015935 ~ small ribosomal subunit	<0.001
	INTERPRO—IPR006032:Ribosomal protein S12/S23	0.011
	PIR_SUPERFAMILY—PIRSF002133:ribosomal protein, S12p/S12a/S23e/organellar S12 types	0.015
1.56	GO Term—GO:0046933 ~ proton-transporting ATP synthase activity, rotational mechanism	<0.001
	Uniprot—KW-0406 ~ Ion transport	<0.001
	Uniprot—KW-0375 ~ Hydrogen ion transport	0.001
1.27	KEGG Pathway—ehx00860:Porphyrin metabolism	0.004
	Uniprot—KW-0149 ~ Chlorophyll biosynthesis	0.016
*Elevated warming (28°C vs. 17°C)*		
Up-regulated proteins		
4.91	GO Term—GO:0005743 ~ mitochondrial inner membrane	<0.001
	Uniprot—KW-0496 ~ Mitochondrion	<0.001
	Uniprot—KW-0999 ~ Mitochondrion inner membrane	<0.001
2.92	Uniprot-KW-0143 ~ Chaperone	<0.001
	GO Term—GO:0006457 ~ protein folding	0.001
	GO Term—GO:0051082 ~ unfolded protein binding	0.011
2.59	KEGG Pathway—ehx01200:Carbon metabolism	<0.001
	KEGG Pathway—ehx01110:Biosynthesis of secondary metabolites	0.004
2.07	KEGG Pathway—ehx00020:Citrate cycle (TCA cycle)	<0.001
	KEGG Pathway—ehx00620:Pyruvate metabolism	0.011
	Uniprot—KW-0670 ~ Pyruvate	0.026
1.79	INTERPRO—IPR020575:Heat shock protein Hsp90, N-terminal	0.005
	INTERPRO—IPR001404:Heat shock protein Hsp90	0.005
	GO Term—GO:0051082 ~ unfolded protein binding	0.011
Down-regulated proteins		
7.04	Uniprot—KW-0934 ~ Plastid	<0.001
	GO Term—GO:0019843 ~ rRNA binding	<0.001
	Uniprot—KW-0699 ~ rRNA-binding	<0.001
3.48	KEGG Pathway—ehx00860:Porphyrin metabolism	<0.001
	GO Term—GO:0006782 ~ protoporphyrinogen IX biosynthetic process	<0.001
	Uniprot—KW-0627 ~ Porphyrin biosynthesis	0.006
3.11	GO Term—GO:0009507 ~ chloroplast	<0.001
	Uniprot—KW-0602 ~ Photosynthesis	<0.001
	GO Term—GO:0016020 ~ membrane	0.002
1.90	GO Term—GO:0015979 ~ photosynthesis	0.002
	GO Term—GO:0042549 ~ photosystem II stabilization	0.005
	GO Term—GO:0009654 ~ photosystem II oxygen evolving complex	0.005
1.19	Uniprot—KW-0375 ~ Hydrogen ion transport	0.006
	GO Term—GO:0046933 ~ proton-transporting ATP synthase activity, rotational mechanism	0.008
	Uniprot—KW-0793 ~ Thylakoid	0.009

*Significant terms are presented from the top 5 enriched clusters (based on enrichment score; “Score,” left-hand column), obtained from lists of significantly up- and down- regulated proteins identified in pair-wise T-tests between the control and warming treatments (*q* ≤ 0.01). Functional terms were identified to be significant when Bonferroni FDR-corrected *p* ≤ 0.05. In cases where >3 terms were found to be significant in each cluster, only the three most significant terms are presented.

#### 3.2.2. Photosynthesis

**Figure 1:**
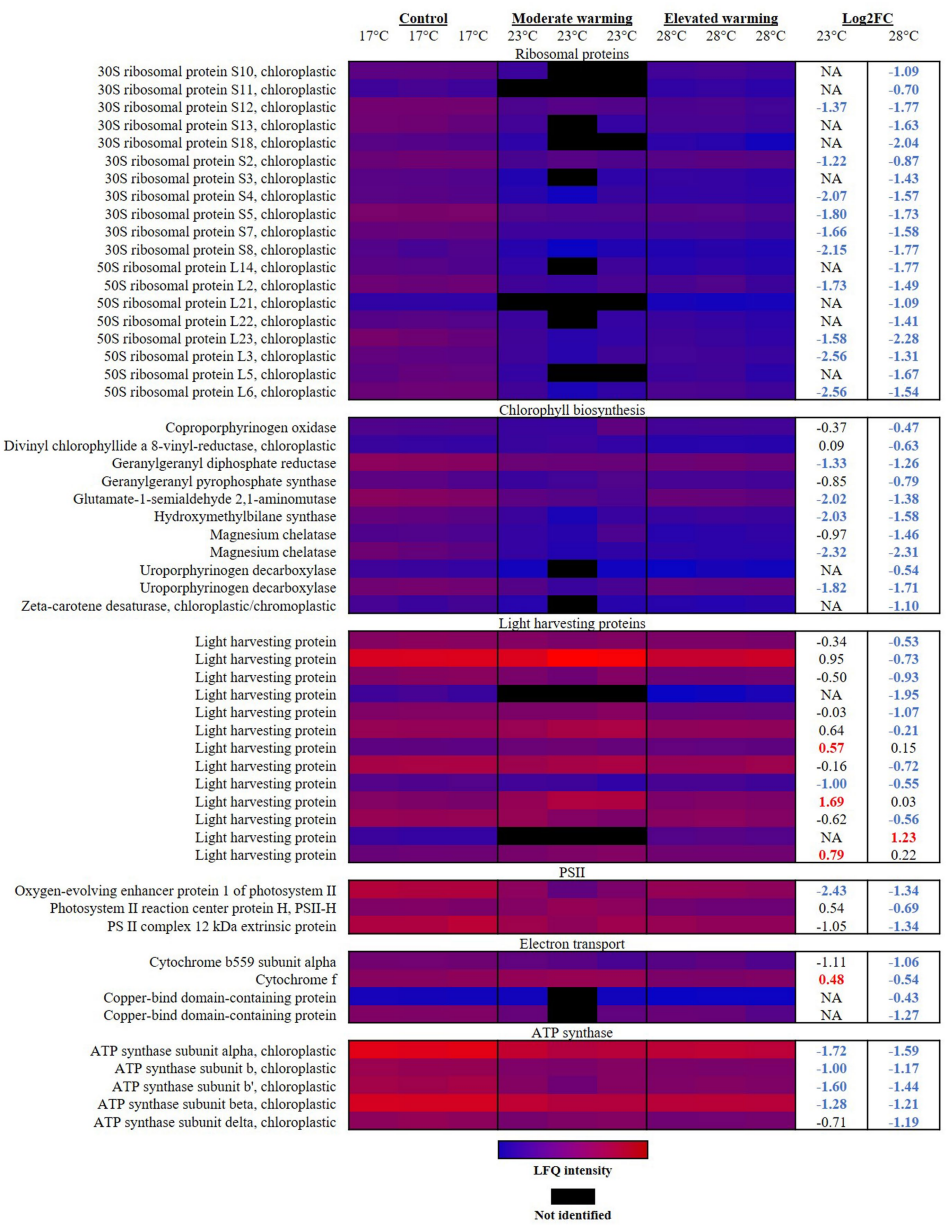
Heatmap displaying photosynthetic proteins significantly altered in abundance under warming (23°C and 28°C) compared to the control (17°C). Relative protein abundances based on raw LFQ intensities are presented in a blue-to-red scale of increasing abundance for each replicate (*n* = 3). Black cells represent where a protein was not identified in the replicate. Respective Log2 fold-changes for each protein in the moderate- and elevated- warming treatment are provided in the right-hand column; significant fold-changes are displayed in bold colored font; whereby red and blue text indicate a relative up- or down- regulation in each treatment compared to the control (*q* ≤ 0.01).

Photosynthesis is considered highly temperature-sensitive (Berry and Björkman, 1980; Yordanov et al., 1986). Typically, photosynthesis displays a negatively-skewed relationship with temperature, following trends observed in phytoplankton growth (Barton et al., 2020). In general, rising temperatures enhance photosynthetic performance due to higher rates of electron transfer and light energy capture (Raven and Geider, 1988). At low temperatures, an uncoupling between energy absorption and consumption can occur, causing photodamage due to excess energy in the photosystem (Fanesi et al., 2016). Past the thermal optima for photosynthesis, heat stress can destabilize and halt normal activity of photosynthetic apparatus and related enzymes, leading to photoinhibition (Allakhverdiev et al., 2008). Hence, at both low and high temperatures, cells must expend energy to protect and repair the inefficient photosystem. In this study, proteomic analysis revealed a dramatic reorganization of photosynthetic machinery in *E. huxleyi* in response to varying temperature (Figure 1), which likely plays a critical role in the energetic balance of this coccolithophore and alteration in growth rate recorded.

Rates of protein translation have been reported as being limited by temperature, and are suggested to represent an effective tool to assess the impact of temperature upon the core metabolism of phytoplankton (Toseland et al., 2013). In response to both moderate- and elevated- warming, enrichment analysis revealed “translation”-related terms to be significantly enriched in down-regulated proteins (Table 2). The vast majority of significantly altered ribosomal proteins were annotated as being chloroplastic, and were significantly down-regulated under warming (Figure 1). Previous work on the diatom, *Thalassiosira pseudonana*, demonstrated that although ribosome production is enhanced at low temperature, the rate of protein synthesis is decreased, believed due to a reduced efficiency of translation (Toseland et al., 2013). It has been proposed that to compensate for this reduction in translation efficiency, cells at low temperatures increase their production of ribosomal proteins (Toseland et al., 2013). Such a process may be one explanation for the relatively high ribosomal protein content observed in *E. huxleyi* cultured at 17°C compared to warming treatments.

In response to warming, *E. huxleyi* appeared to down-regulate chlorophyll biosynthesis. Declines in protein abundance were identified throughout the chlorophyll biosynthetic pathway in cultures grown under both warming treatments (Figure 1). Similar declines in chlorophyll content and biosynthesis have been recorded in marine diatoms in response to warming (Thangaraj and Sun, 2021; Cheng et al., 2022). Land plants have also been reported to reduce chlorophyll synthesis during heat stress (Dutta et al., 2009; Efeoglu and Terzioglu, 2009), suggesting that this may be a conserved feature of the photosynthetic response to temperature. A total of 11 proteins involved in the chlorophyll biosynthetic pathway were identified to significantly decrease in abundance across the two warmed treatments when compared to the control (*q* ≤ 0.01). For example, glutamate-1-semialdehyde 2,1-aminomutase (R1DH70), which plays a key role in the conversion of glutamate to porphyrin precursors during the first stage of chlorophyll synthesis (Pugh et al., 1991), displayed a-2.02 FC and-1.38 FC decline in response to growth at moderate- and elevated- warming, respectively (*q* ≤ 0.01). In both warming treatments, greatest alteration in protein abundance in relation to chlorophyll biosynthesis was in magnesium chelatase (R1F8J6), which displayed an almost identical −2.30 FC decline relative to the control (*q* ≤ 0.01). This protein plays a critical role in both heme and chlorophyll synthesis, as up to this point both compounds share a common precursor pathway. Magnesium chelatase catalyses the addition of Mg^2+^ into protoporphyrin IX, the product of which continues to produce chlorophyll. Addition of Fe^2+^ at this stage would alternatively produce heme (Walker and Willows, 1997). The protein which catalyses this second reaction (Ferrochetalase; Walker and Willows, 1997) was not identified during proteomic analysis, so we are unable to assess whether heme synthesis was also affected by temperature.

Light harvesting proteins (LHPs) are utilized in photosynthesis to capture and direct light energy toward the reaction centers of photosystems I (PSI) and II (PSII) (Curutchet et al., 2011). In this study, we observed that at relatively low temperatures (17°C), below the optimum for growth, *E. huxleyi* contains a high abundance of LHPs relative to cultures grown at higher temperatures (28°C). Indeed, in response to elevated warming, a total of nine LHPs were significantly down-regulated compared to the control, representing a FC of −0.53 to −1.95 (*q* ≤ 0.01) (Figure 1). In contrast, little significant difference was recorded in the abundance of LHPs between the control and moderate warming treatment, where growth rates were highest. Rather, in this case three LHPs were observed to significantly increase in abundance (Figure 1). While the physical process of light absorption is not typically considered to be affected by temperature, rates of excitation energy transfer and photosynthetic electron transport are believed to be limited at lower temperature (Geider, 1987; Raven and Geider, 1988; Falkowski and Raven, 1997; Ensminger et al., 2006). This likely explains the relative up-regulation of LHPs in the 17°C control, whereby the light harvesting apparatus is maximized to ensure sufficient capture of light energy to drive the less efficient photosynthetic electron chain. In contrast, at elevated temperatures, LHPs may be down-regulated to reduce light energy capture in a heat-stressed photosystem.

PSII is believed to be particularly susceptible to temperature (Berry and Björkman, 1980; Srivastava et al., 1997). Here, damages to the reaction center and oxygen-evolving complex (OEC) of PSII are reported to be a key feature of heat stress, caused by a dissociation of Ca^2+^, Mn^2+^ and Cl^−^ cofactors from the PSII complex and release of extrinsic polypeptides from the thylakoid membrane (Berry and Björkman, 1980; Havaux, 1993; Salvucci et al., 2001; Wise et al., 2004). Coupled with the down-regulation of LHPs at high temperature, the PSII reaction center protein H (Q4G3C2) was also significantly reduced −0.69 FC under elevated warming compared to the control and -1.23 FC compared to the moderate warming treatment (*q* ≤ 0.01). This protein was on average up-regulated under moderate warming, likely improving energy transfer in PSII (Figure 1). In addition, cytochrome b559 subunit alpha (Q4G380) and cytochrome f (Q4G3D7), involved in photosynthetic electron transport were also reduced under elevated warming (*q* ≤ 0.01), providing further evidence of heat stress (Mathur et al., 2014). In contrast, cytochrome f was significantly up-regulated 0.48 and 1.02 FC under moderate warming treatment versus the control and elevated warming treatment, respectively (*q* ≤ 0.01). The OEC oxidizes water to produce molecular oxygen via the sequential removal of four electrons (Bricker et al., 2012), in doing so, it produces the proton gradient required to drive ATP generation by ATP synthase. Enrichment analysis revealed that under elevated warming, the OEC was significantly reduced compared to the control (Table 2). Two proteins were identified to drive this trend; the oxygen-evolving enhancer protein 1 of PSII (R1D369) and the PSII complex 12 kDa extrinsic protein (R1BY69) (Figure 1). These proteins function to enhance the water splitting reaction and the structural stability of PSII (Bricker et al., 2012). While, the OEC was not identified by enrichment analysis when examining significant proteins in the moderate warming treatment, the oxygen-evolving enhancer protein 1 of PSII was found to display one of the greatest fold-changes in this treatment (−2.43 FC, *q* ≤ 0.01), suggesting that the decrease in abundance of OEC proteins is temperature-dependent.

As described, the OEC produces the proton gradient required for ATP generation via ATP synthase located within the chloroplast thylakoid membrane. Enrichment analysis identified chloroplastic ATP synthase-related proteins to be down-regulated in warming treatments relative to the 17°C control (Table 2). Five ATP synthase subunits (Q4G3C8; Q4G397; Q4G398; R1DKU4; Q4G399; Q4G3A0) were significantly reduced in the elevated warming treatment when compared to the control (−1.17–1.59 FC), four of which were also significantly reduced under moderate warming (−0.71–1.72 FC) (*q* ≤ 0.01) (Figure 1). Further investigation is required to fully understand the implications of this down-regulation of ATP synthase, where measurements of ATP production will be particularly beneficial.

Generally, PSI is believed to more resilient to heat stress than PSII, rather research has shown that temperature rises in the range that are detrimental to PSII can enhance the rate of PSI-mediated electron transport (Ivanov et al., 2017). In line with this, in our work, four proteins belonging to PSI were identified during analysis, however no significant alteration in their abundance in response to warming was recorded. Despite the proposed enhancement of PSI electron transport activity with increasing temperature, two copper-bind domain-containing proteins (R1EBM1 and R1B8S7), belonging to the plastocyanin family, were significantly decreased −0.43 and − 1.27 FC in the elevated warming treatment compared to the control (*q* ≤ 0.05). Plastocyanin functions in the transfer of electrons from PSI to ferredoxin (Ivanov et al., 2017). The reduction of plastocyanin family proteins with increasing temperature may indicate a reduction in PSI electron transport activity or reduced plastocyanin requirement at high temperature compared to control cultures. However, whether this arises as a consequence of thermal impacts on PSI or due to an increase in cyclic PSI activity at low temperatures when electron transport efficiencies are reduced is not clear. Further investigation utilizing pulse amplitude modulated (PAM) fluorometry would be beneficial to resolve these findings.

In summary, it appears that when growing at relatively low temperature (17°C), *E. huxleyi* invests in photosynthetic machinery to compensate for the reduced efficiency of light energy capture and its transport through the photosynthetic electron chain. This is evident by increased chloroplastic translation in control cultures grown at 17°C, an up-regulation of chlorophyll biosynthesis, and an increase in abundance of LHPs. Notably, to utilize this increase in light harvesting apparatus and make-up for the reduction in electron transfer efficiency, *E. huxleyi* grown at low temperature appears to up-regulate the OEC and increase production of ATP synthase, to maximize photosynthetic ATP production. However, this investment in high-cost photosynthetic machinery (Halsey and Jones, 2015), appears to compromise growth and hence the incorporation of inorganic carbon into biomass. In contrast, at elevated temperatures (28°C) *E. huxleyi* down-regulates its capability to harvest light energy by reducing the abundance of LHPs and components of PSII, perhaps as a photoprotective response. Heat stress causes damage to PSII which in-line with previous research is associated with damages to the PSII reaction center and OEC. Growth is still maintained at a higher rate than at low temperature (17°C), perhaps due to the relative improvement of light capture and electron transport with increasing temperature and other metabolic alterations associated with temperature, discussed below. We can therefore suggest that at temperatures closest to the optimum for growth, the efficiency of the light harvesting system is enhanced and a lesser extent of heat stress is experienced, therefore requiring less investment in photosynthetic machinery and repair mechanisms. As a result, cells are able to maximize energy for growth and incorporation of photosynthetic carbon into biomass. If conserved across phytoplankton groups, we can predict that increases in temperature within the sub-optimal to optimal thermal range for photosynthesis will enhance carbon sequestration by phytoplankton.

#### 3.2.3. Respiration and carbon metabolism

**Figure 2:**
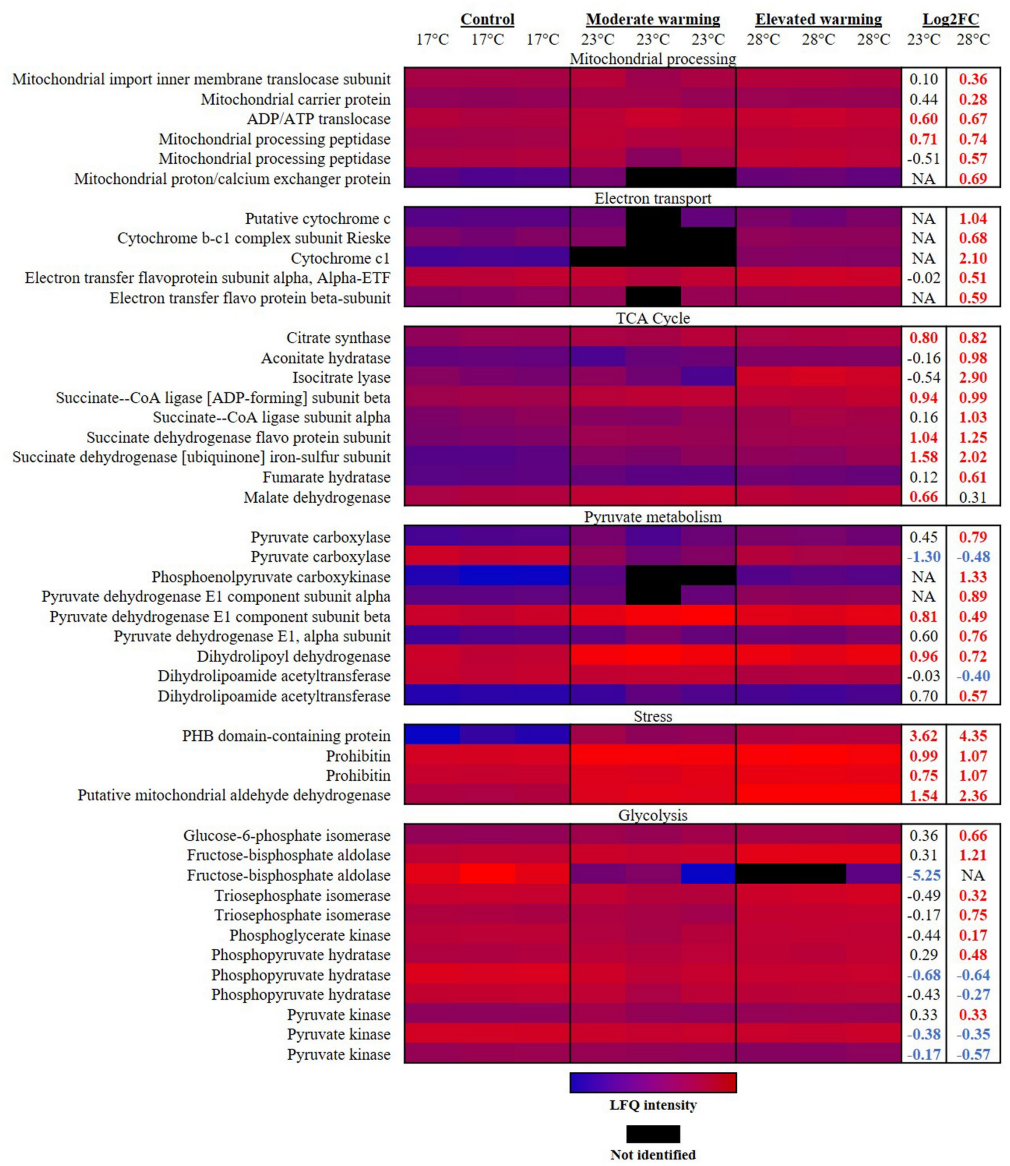
Heatmap displaying respiratory proteins significantly altered in abundance under warming (23°C and 28°C) compared to the control (17°C). Relative protein abundances based on raw LFQ intensities are presented in a blue-to-red scale of increasing abundance for each replicate (*n* = 3). Black cells represent where a protein was not identified in the replicate. Respective Log2 fold-changes for each protein in the moderate- and elevated- warming treatment are provided in the right-hand column; significant fold-changes are displayed in bold colored font; whereby red and blue text indicate a relative up- or down- regulation in each treatment compared to the control (*q* ≤ 0.01).

It has previously been reported that as temperatures increase, rates of respiration rise at a higher rate than photosynthesis (Barton et al., 2020). In the short term, this process leads to a greater proportion of photosynthetic carbon being respired and is hence less available for growth, described as a reduction in carbon-use-efficiency (Gifford, 2003; Allison et al., 2010; Padfield et al., 2016; Barton et al., 2020). In line with this, terms related to the mitochondrion were identified as significantly enriched in analysis of up-regulated proteins identified in warming treatments (Table 2). Highest mitochondrial activity under elevated warming was evident by the significant rises in abundance of proteins involved in mitochondrial processing (Figure 2). The mitochondria are the primary base of carbon metabolism within the cell. Herein, proteomic analysis revealed a remarkable alteration to carbon metabolism in cultures acclimated to elevated temperatures (28°C), which centered around the tricarboxylic acid (TCA) cycle. Typically, the TCA cycle is a major component of energy production, producing ATP, CO_2_ and the redox cofactors required to drive the mitochondrial electron transport chain (ETC) (Halsey and Jones, 2015). The mitochondrial ETC produces the majority of ATP in eukaryotic cells (Jardim-Messeder et al., 2015). Components of the mitochondrial ETC were found to be significantly up-regulated with increasing temperature relative to the control (Figure 2). For example, the succinate dehydrogenase iron–sulfur subunit (R1F8P8) and succinate dehydrogenase flavoprotein subunit (R1EUN2) of complex II both displayed a significant temperature-wise increase representing a FC of 1.58 and 2.02 (R1F8P8), and 1.04 and 1.25 (R1EUN2) in the moderate- and elevated- warming treatments, respectively (*q* ≤ 0.01). Such results suggest that ATP production via the respiratory ETC activity was enhanced in *E. huxleyi* as temperatures increased. This is supported by a significant increase in abundance of the mitochondrial ADP/ATP translocase (0.60–0.67 FC; R1D5S6) recorded in warming treatments (*q* ≤ 0.01).

*Emiliania huxleyi* up-regulated a number of components of the TCA cycle when acclimated to growth under elevated warming (Figure 2). Many of these components were also significantly raised under moderate warming compared to the control. Similar findings have been reported during proteomic analysis of the marine diatom, *Skeletonema dohrnii*, as well as during transcriptomic analyses on coccolithophore, prymnesiophyte and dinoflagellate taxa in response to warming combined with acidification (Cheng et al., 2022; Liang et al., 2023; Thangaraj et al., 2023; Zhang et al., 2023). In *E. huxleyi*, this process appears fuelled by an increase in acetyl-CoA production via pyruvate and acetoacetyl CoA, catalyzed by the pyruvate dehydrogenase complex and acetyl-CoA acetyltransferase (R1FQ80), each significantly up-regulated in the elevated warming treatment relative to the control (*q* ≤ 0.01). Expression of the latter has previously been recorded to increase in the coccolithophore *Chrysotila dentata* following exposure to combined warming and acidification treatments (Thangaraj et al., 2023).

**Figure 3:**
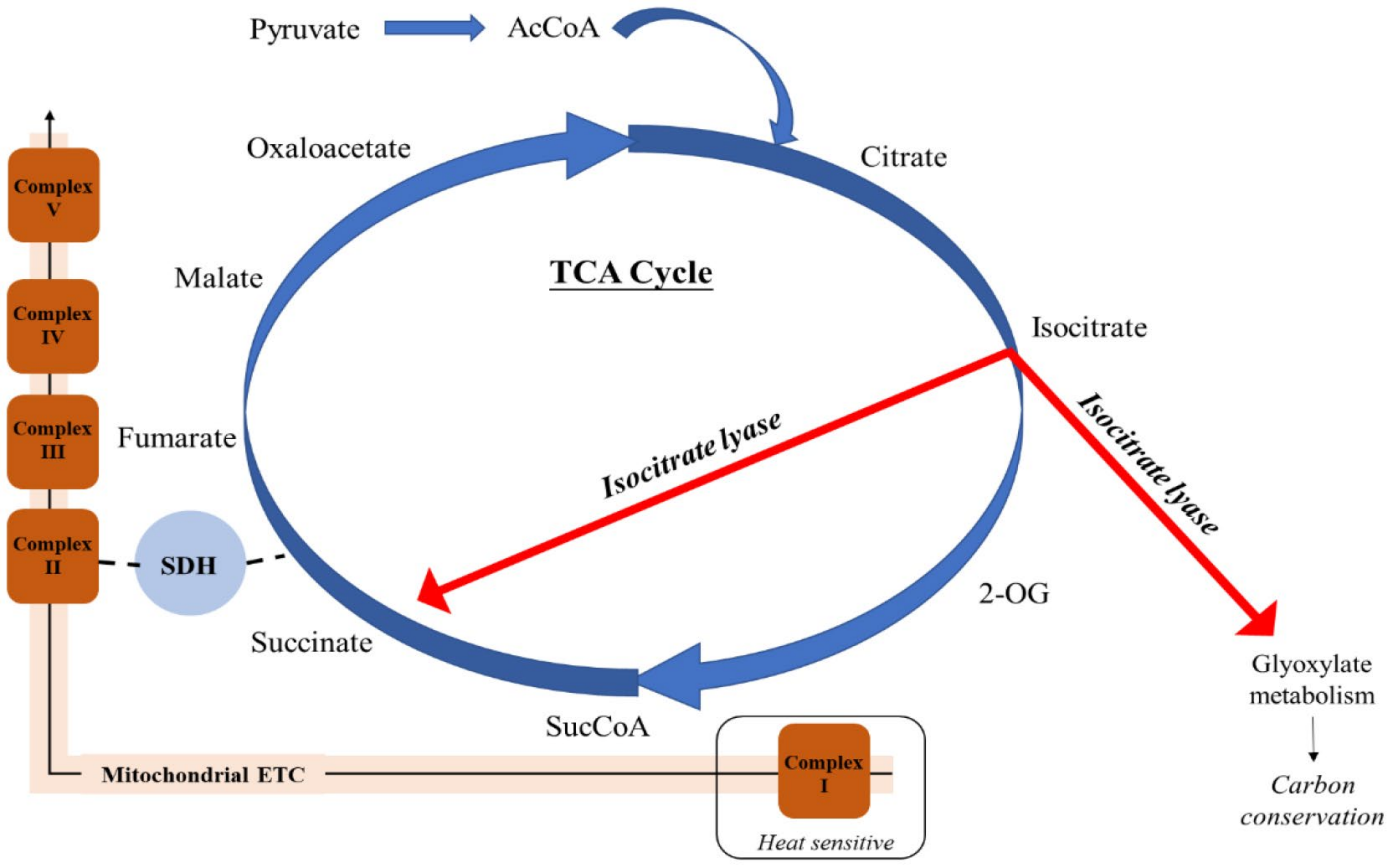
Employment of isocitrate lyase to bypass the heat-sensitive respiratory Complex I of the mitochondrial electron transport chain and conserve carbon in *Emiliania huxleyi* acclimated to elevated warming. Acetyl CoA (AcCoA); 2-Oxoglutarate (2-OG); Succinyl CoA (SucCoA); Succinate dehydrogenase (SDH).

A key feature identified during analysis of respiratory proteins was that when growing at elevated temperatures, *E. huxleyi* appeared to utilize isocitrate lyase (R1C144) to by-pass several stages in the TCA cycle, producing succinate and glyoxylate (Figure 3). In this manner, isocitrate lyase which displayed a 2.90 FC and 3.44 FC increase compared to the control and moderate warming treatments, respectively (*q* ≤ 0.01), redirects a proportion of acetyl CoA toward the TCA and glyoxylate cycles rather than into the fatty acid (FA) biosynthetic pathway, creating a net gain in carbon (Dolan and Welch, 2018). This process is believed to be a feature of a carbon-conservation strategy as carbon substrates are recycled and can be used to build cell biomass (Beier et al., 2015; Dolan and Welch, 2018). Related to this, we observed a 1.07 FC up- regulation of glycine hydroxymethlytransferase (R1FJG9) under elevated warming compared to the control (*q* ≤ 0.01). In photosynthesising cells, glycine hydroxymethyltransferase catalyses the conversion of glycine to serine within the photorespiratory cycle (Kern et al., 2011). In the cytosol, serine reacts with glyoxylate to form hydroxypyruvate, a precursor of glycerate-3-phosphate (Kern et al., 2011). This process is part of the 2-phosphoglycolate cycle of photorespiration, the product formed by the oxygenase reaction of RuBisCO (Kern et al., 2011). The up-regulation of the serine-forming reaction observed at relatively high temperature within the elevated warming treatment (28°C) may indicate an increase in RuBisCO oxygenase activity as dissolved CO_2_ concentrations decrease relative to O_2_ as temperatures rise (Zeebe and Wolf-Gladrow, 2007; Griffiths et al., 2017). Enhancing serine production in this manner may display use of the 2-phosphoglycolate cycle as an alternative pathway for glycerate-3-phosphate generation as RuBisCO carboxylation activity is compromised. Doing so comes at an energetic cost and reduction in carbon fixation (Heureux et al., 2017). Alternatively, serine may be exported to the cytosol as a one-carbon metabolite for other biosynthetic pathways (Bauwe and Kolukisaoglu, 2003).

Isocitrate lyase also catalyses the production of succinate, which is additionally formed from succinyl-CoA by the activity of succinate-CoA ligase, producing ATP or GTP (Johnson et al., 1998). This enzyme was significantly more abundant in both warming treatments compared to the control (0.94–1.03 FC; *q* ≤ 0.01). Succinate contributes to both the mitochondrial ETC and TCA cycle, and is believed to enable a “shortcut” to oxidative ATP production (Figure 3) (Giorgi-Coll et al., 2017). It has been described that succinate enables the mitochondrial ETC to bypass complex I, reported to be particularly susceptible to stress (Berg et al., 2002; Rich and Marechal, 2010). As a result, succinate is able to sustain ATP production in damaged mitochondria (Giorgi-Coll et al., 2017). Further support of the increased utilization of succinate in this manner under warming conditions is revealed by identification of proteins related to mitochondrial stress (Figure 2). Prohibitin (PHB) has been suggested to play a role in the biogenesis of mitochondria and protection against mitochondrial oxidative stress and aging in plants (Ahn et al., 2006). Herein, one of the most up-regulated proteins in both warming treatments relative to the control was the PHB-containing protein (R1DW75), which displayed a 3.62 FC and 4.35 FC in the moderate- and elevated- warming treatments, respectively (*q* ≤ 0.01). A further two PHBs (R1FPT0 and R1EXU2) were identified as being significantly more abundant under warming treatments, increasing 0.75–1.07 FC (*q* ≤ 0.01). Warming additionally caused a respective 1.54 and 2.36 FC increase in abundance of mitochondrial aldehyde dehydrogenase (R1EIW7) in the moderate- and elevated- warming treatments relative to the control (*q* ≤ 0.01). This enzyme, which was significantly more abundant under elevated- compared to moderate- warming, operates in the homeostasis of toxic aldehydes which can accumulate following the production of ROS (Tola et al., 2020), routinely generated by the ETC. When acclimated to growth at increased temperature *E. huxleyi* utilizes succinate-mediated oxidative ATP production to mitigate thermal stress exerted upon the mitochondria.

Alongside its role in mitochondrial electron transport, succinate dehydrogenase links the ETC and TCA cycle by catalyzing the oxidation of succinate to fumarate, producing the electrons which are utilized by the ETC (Huang and Millar, 2013). As in our work, an up-regulation of succinate dehydrogenase has previously also been recorded in the diatom *S. dohrnii* in response to elevated warming (Cheng et al., 2022). In the TCA cycle fumarate is later converted to oxaloacetate via malate. By action of phosphoenolpyruvate carboxykinase (PEPCK), oxaloacetate is converted to phosphenolpyruvate, producing CO_2_. In our study, a significant 1.33 FC up-regulation of PEPCK (R1EG52) was observed in the elevated warming treatment compared to the control (*q* ≤ 0.01). Due to the relatively poor affinity of its RuBisCO to CO_2_, *E. huxleyi* is believed to utilize carbon concentration mechanisms (Stojkovic et al., 2013). PEPCK has been proposed to be a CO_2_ concentrating mechanism in *E. huxleyi* (Wahlund et al., 2004), hence its up-regulation at relatively high temperature may increase the CO_2_ available to RuBisCO and in doing so increase its carboxylation relative to oxygenase activity, described above. A decline in RuBisCO carboxylation activity appeared evident at elevated temperatures due to a − 0.78 FC decline in phosphoribulokinase (R1E1E6; *q* ≤ 0.01) at 28°C which regenerates ribulose 1,5-bisphosphate, the substrate for carbon fixation. However, no alteration in abundance of RuBisCO was recorded in either warming treatment. Three additional enzymes involved in the Calvin Benson cycle were found to significantly alter in abundance in response to warming in *E. huxleyi*. Transketolase is responsible for the interconversion of sedoheptulose-7-phosphate and glyceraldehyde 3-phosphate to ribose-5-phosphate and xylulose 5-phosphate, in addition to the reversible conversion of fructose 6-phosphate and glyceraldehyde 3-phosphate to xylulose 5-phosphate and erythrose 4-phosphate (Suzuki et al., 2017). Abundance of transketolase (R1DMT8) was significantly reduced −1.00 FC and −0.65 FC in response to moderate- and elevated- warming compared to the control (*q* ≤ 0.01). Related to this, abundance of ribose-5-phosphate isomerase (R1FK83) was also reduced −0.45 to −0.58 FC under warming (*q* ≤ 0.01). Taken together, these results suggest a reduction in the direction of carbon metabolism toward ribulose 1,5-bisphosphate generation via ribulose 5-phosphate in warming treatments relative to the control. Additionally, ribulose-phosphate 3-epimerase (R1CZ77) was increased 1.18 FC under elevated warming, compared to the control (*q* ≤ 0.01). This enzyme catalyses the interconversion between xylulose 5-phosphate and ribulose 5-phosphate (Teige et al., 1998). Such alterations may arise due to a greater efficiency of photosynthetic and carbon fixation reactions at higher temperature, as well as greater energy availability demonstrated by enhanced growth rates, thus freeing up carbon products to be directed toward alternative metabolic pathways.

A number of proteins involved in glycolysis were significantly up-regulated under elevated warming (Figure 2), likely sustaining the rise in TCA cycle activity described above by producing pyruvate. Indeed, proteins involved in pyruvate metabolism were also generally more abundant in this treatment (Table 2; Figure 2). Triosephosphate isomerase is a key enzyme of the glycolytic pathway, which catalyses the interconversion between glyceraldehyde-3-phosphate and dihydroxyacetone phosphate (Zaffagnini et al., 2014). This protein (R1BGG4; R1CNI7) was significantly raised in the elevated warming treatment relative to both the control and moderate warming treatments (*q* ≤ 0.01). Related to this, abundance of glyceraldehyde-3-phosphate dehydrogenase (R1DPS6) was recorded to be 0.67 FC higher under elevated- compared to moderate- warming (*q* ≤ 0.01), and on average more abundant than the control. These results suggest an increase in glycolytic activity in the elevated warming treatment. However, in some cases trends in abundance of glycolytic proteins were observed to vary under elevated warming, here additional study via metabolomics would be greatly beneficial to resolve the relative rates of glycolytic activity at each temperature. Generally, glycolytic proteins were less abundant under moderate warming compared to the control, likely owing to improved photosynthetic efficiency and hence surplus energy which could be directed toward biomass production. Notably, the fructose-bisphosphate aldolase (R1CXH4) was recorded to significantly decrease −5.25 FC in abundance when comparing the control and moderate warming treatments (*q* ≤ 0.01). Despite the apparent increase in carbon directed toward the TCA and glycolysis cycles in the elevated warming treatment, a ~ 1 FC increase in abundance of UTP-glucose-1-phosphate uridylyltransferase (R1F553) was recorded under both moderate- and elevated- warming conditions (*q* ≤ 0.05). This enzyme catalyses the production of the universal activated form of glucose, UDP-glucose (Roeben et al., 2006), likely playing a role in fuelling the higher growth rates recorded under warming compared to control cultures.

It has been reported that under stress conditions coccolithophores utilize lipids as an alternative energy reserve and source of carbon for cellular metabolism (Fernandez et al., 1994; Thangaraj et al., 2023). The enzyme, glycerol-3-phosphate dehydrogenase plays a key role linking carbohydrate and lipid metabolism by converting dihydroxyacetone phosphate produced by the action of triosephosphate isomerase to L-glycerol-3-phosphate (Yao et al., 2014). Abundance of glycerol-3-phosphate dehydrogenase (R1DG23) was significantly raised ~0.55 FC under elevated warming compared to both the control and moderate warming treatments, respectively (*q* ≤ 0.01), suggesting an increase in lipid production in this treatment, perhaps in response to heat stress. Alongside altered TCA cycle and glycolytic activity in *E. huxleyi* in warmed treatments, significant alteration to FA metabolism was also recorded. While long-chain acyl-CoA synthetase (R1CXA2) was raised 1.84 FC and 2.35 FC in the moderate- and elevated- warming treatments compared to the control, respectively (*q* ≤ 0.01), a number of proteins involved in FA degradation were found to be significantly raised under both moderate- and elevated- warming, with a greater number recorded in the elevated warming treatment. Similarly, previous work on the coccolithophore, *C. dentata*, displayed up- and down- regulation in expression of transcripts relating to FA biosynthesis and degradation in response to ocean warming and acidification (Thangaraj et al., 2023). Herein, greatest alteration in FA degradation proteins was seen in abundance of the long-chain specific acyl-CoA dehydrogenase (R1CPW4) under elevated warming, which was significantly raised 1.38 FC compared to the control (*q* ≤ 0.01). Abundance of enoyl-CoA hydratase, which catalyses the second step of the FA beta-oxidation pathway, was significantly increased under both moderate- (1.22 FC) and elevated- (1.05 FC) warming (*q* ≤ 0.01). Transcripts related to this protein were also significantly up-regulated in *C. dentata* exposed to warming and acidification (Thangaraj et al., 2023). An increase in FA biosynthesis in the two warming treatments relative to the control is largely expected given their higher energy surplus, indicated by higher rates of growth. The up-regulation of FA synthesis and degradation observed in the elevated warming treatment may arise from a greater requirement for acetyl-CoA to fuel the TCA cycle and utilization of FAs as an alternative carbon source under heat stress, as described above. This is supported by a 1.67 FC reduction in abundance of the acyl carrier protein (R1D0Y2) under elevated warming compared to the control (*q* ≤ 0.01) and use of isocitrate lyase to direct acetyl-CoA toward the TCA cycle. In previous work on the phytoplankton response to warming, *Phaeocystis globosa* was found to direct energy toward the TCA rather than toward FA production under combined exposure to warming and acidification (Liang et al., 2023). Notably, abundance of polyketide synthase (R1EQS3) appeared reduced with temperature, declining in abundance −1.38 FC under moderate and −1.87 FC under elevated warming, relative to the control (*q* ≤ 0.01).

Taken together, these results reveal that *E. huxleyi* is able to fine tune its metabolism to maximize ATP generation, under warming. At lower temperatures, protein abundance appears directed toward photosynthetic machinery, while at elevated temperatures, this coccolithophore modifies its carbon metabolism to sustain ATP production and maximize growth under varying thermal conditions. Notably, such changes to photosynthetic and respiratory activity had no impact upon the expression of RuBisCO itself, however its performance may have altered with temperature (Hermida-Carrera et al., 2016). Metabolomic analysis has been utilized to examine alterations in metabolite production along a number of cycles involved in carbon metabolism in *Arabidopsis* under elevated temperature (Wang et al., 2020), as well as to assess redox homeostasis in green algae under stress (Zhu et al., 2021). Additional study via metabolomics would allow us to confirm and quantitively assess alterations in cellular carbon fluxes and the implications of the metabolic modifications recorded in *E. huxleyi*. In particular, quantifying the amount of succinate and glyoxylate in elevated warming treatments would allow us to confirm the use of isocitrate lyase as a mitigatory mechanism against mitochondrial heat damage, described above. Such research offers great scope for future work. It is clear that an increase in temperature incurs an enhanced level of stress upon the mitochondria of *E. huxleyi*, whereby increases in the generation of toxic compounds such as ROS and aldehydes induces stress response and repair pathways. Given that *E. huxleyi* maintained significantly higher growth rates despite this stress in both warming treatments compared to the 17°C control, it appears that the induced response is effective to ensure the mitochondria is able to continue functioning effectively. The reduced growth observed in the elevated- versus the moderate- warming treatment likely results from the increasing cost of repair and reduction in efficiency of carbon metabolism at high temperature. It remains to be examined at which point temperature exerts sufficient stress to completely overwhelm the mechanisms observed. It is likely that ultimately the cost of repair will constrain growth at extreme temperature.

#### 3.2.4. Protein synthesis and processing

Increasing temperature is recorded to enhance the efficiency of protein synthesis in eukaryotic phytoplankton, while simultaneously reducing ribosomal content (Toseland et al., 2013; Thangaraj et al., 2023). As such, phytoplankton are reported to require fewer ribosomes to produce an equal amount of cellular protein when growing at higher temperatures (Toseland et al., 2013). Accordingly, both the ribosome recycling factor (−1.01 FC; R1F821) and translation initiation factor IF-2 (−1.51 FC; R1D2Z9) were significantly reduced under elevated warming (*q* ≤ 0.01), and *E. huxleyi* displayed a significant down-regulation of ribosomal proteins under warming treatments relative to the control (section 3.1).

**Figure 4:**
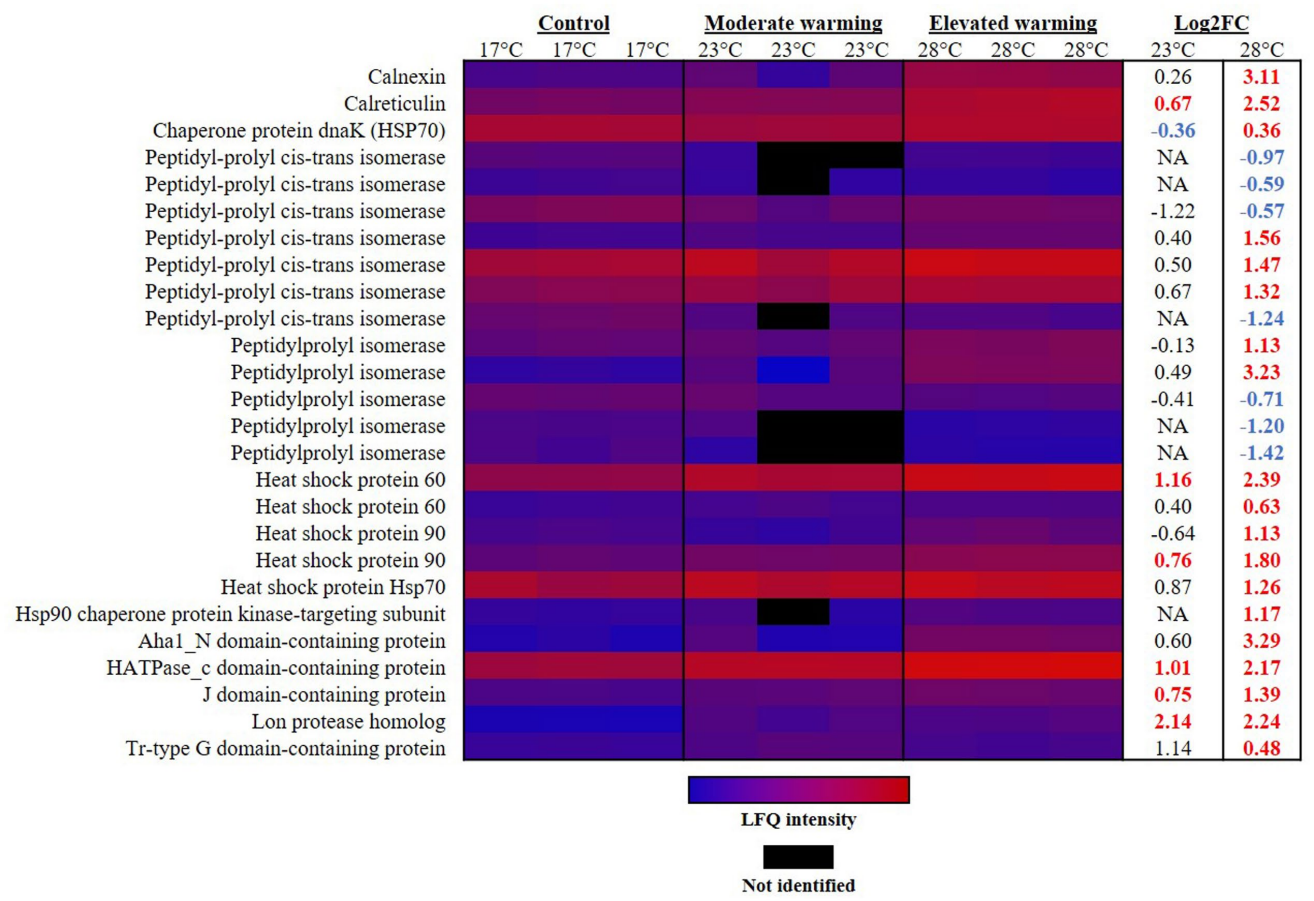
Heatmap displaying proteins involved in protein processing which were significantly altered in abundance under warming (23°C and 28°C) compared to the control (17°C). Relative protein abundances based on raw LFQ intensities are presented in a blue-to-red scale of increasing abundance for each replicate (*n* = 3). Black cells represent where a protein was not identified in the replicate. Respective Log2 fold-changes for each protein in the moderate- and elevated- warming treatment are provided in the right-hand column; significant fold-changes are displayed in bold colored font; whereby red and blue text indicate a relative up- or down- regulation in each treatment compared to the control (*q* ≤ 0.01).

In-line with earlier reports, in this study protein processing, and hence synthesis, appeared enhanced under warming in *E. huxleyi*. Protein processing occurs largely in the endoplasmic reticulum (ER). Two proteins that play an important role in this compartment are calreticulin and calnexin. Calreticulin is a highly conserved Ca-binding protein in the ER lumen which plays a key role in Ca homeostasis and storage, and the correct conformation of proteins (Michalak et al., 1999). This protein is reported to display high themostability (Wijeyesakere et al., 2011), and is considered to be induced by environmental stress, such as heat-stress in corals (Yuyama et al., 2012; Dunlap et al., 2013). Accordingly, calreticulin (R1D6Y1) was significantly raised under both warming treatments, although to a higher extent under elevated warming, representing a FC of 0.67 (23°C) and 2.52 (28°C) in each treatment, respectively, (*q* ≤ 0.01) (Figure 4). Calnexin is a homolog of calreticulin and also functions to ensure correct protein folding (Bergeron et al., 1994). This protein was significantly raised under elevated warming only and displayed one of the highest rates of FC, 3.11 (*q* ≤ 0.01). The relative up-regulation of calreticulin and calnexin in the elevated warming treatment, suggests an increased requirement for protein processing as rates of protein synthesis increase and cells experience heat stress, likely due to damages to protein stability. Interestingly, in heat stressed plants, ROS production has been reported to increase the concentration of Ca in the cytosol, which subsequently enters organelles such as the mitochondria (Rikhvanov et al., 2014; Sharma et al., 2022). Due to its role in cell signaling, this influx of Ca may compromise cell signaling in a range of cellular compartments. Therefore, an alternative function of the up-regulation of calreticulin and calnexin observed under warming recorded herein may be to reduce cytosolic Ca concentrations and thus mitigate any disruption to cell signaling caused by its entry into nearby organelles.

Peptidylprolyl isomerases appear in both prokaryotic and eukaryotic cells. These foldase proteins play a role in the folding of newly synthesized proteins (Shaw, 2002; Lin et al., 2019). In line with alterations in protein processing under warming, abundance of peptidylprolyl isomerase displayed significant alterations in abundance. Notably, significant differences were only recorded in the elevated warming treatment, however a number of PPIases were not identified in cultures acclimated to moderate warming. Five PPIases were raised 1.13–3.23 FC in the elevated warming treatment, while 7 appeared significantly down-regulated (*q* ≤ 0.01) (Figure 4). Such results provide further evidence to disruption to normal cell function when exposed to high temperatures.

Heat shock proteins (Hsps) are ATP-dependent molecular chaperones, considered the first line of defense against cellular stress (Dubrez et al., 2020). These proteins, which act to support protein stabilization, folding, and transport, as well as contributing to cell regulatory processes are highly conserved across both animal and plant species (Lindquist and Craig, 1988; Vierling, 1991; Dubrez et al., 2020). Hsps are present in the cell under normal conditions but are up-regulated in response to a number of stressors including, temperature, heavy metal and oxidative stress (Li et al., 2009; Pratt et al., 2010; Morales et al., 2011). In our work, we identified a number of Hsps to be significantly increased under warming treatments compared to the control, providing evidence of heat stress (Figure 4). As expected, this was most apparent under elevated warming. Here, the protein displaying highest rates of FC was the Aha1_N domain-containing protein (R1C1F8), which increased 3.29 FC in the elevated warming treatment compared to the control (*q* ≤ 0.01). This protein was annotated with the “ATPase activator activity” [GO:0001671] and “Hsp90 protein binding” [GO:0051879], suggesting its role in the activation of Hsp90. Hsp90 represents the most widely conserved and abundant Hsp (Zhang et al., 2019). Two Hsp90 proteins were identified in this study (R1CAZ6 and R1BIY8), both of which were significantly more abundant in cultures acclimated to elevated warming compared to the control, representing a FC of 1.80 and 1.13, respectively, (*q* ≤ 0.01). The former was also significantly more abundant in the moderate warming treatment, however to a lesser extent (0.76 FC; *q* ≤ 0.01). Hsp60 (R1D6F2), represented the most up-regulated Hsp in either warming treatment. When compared to the control, this protein increased 1.16 FC and 2.39 FC in the moderate- and elevated- warming treatments, respectively, and was significantly more abundant under elevated- compared to moderate- warming (*q* ≤ 0.01). The regulation of Hsps is species-specific and is influenced by local environmental conditions. For example, Hsps are up-regulated in arctic fish at temperatures as low as 5°C, while their activation does not occur until temperatures of ~100°C in thermophilic bacteria (Parsell and Lindquist, 1994; Feder and Hofmann, 1999; Sorensen et al., 2003). Given that Hsps are ATP-dependent, their energetic cost will inevitably be influenced by the threshold temperature at which their expression is induced. Here, it is likely that those species that are more sensitive to increases in temperature will experience a greater cost of repair, and hence their growth will be limited. Use of energy in this fashion will likely have a negative impact upon carbon fixation in phytoplankton experiencing thermal stress. It is possible that the enhancement of mitochondrial investment observed in cultures acclimated to elevated warming may be a necessary consequence of the increased energetic demand for repair and protein synthesis, exacerbated by declines in photosynthetic apparatus, hence constraining growth. Indeed, it has been suggested that up-regulation of the TCA cycle under warming may provide the energy required to fuel increases in protein synthesis (Zhang et al., 2023). Long-term adaptation to growth at higher temperature may reduce the energetic costs associated with repair, thus freeing up energy for growth. Evidence from longer-term laboratory assessment of phytoplankton adaptation to warming suggests that such adjustments to metabolism are likely (Padfield et al., 2016; Barton et al., 2020), but evaluating which biochemical mechanisms facilitate such a response provide an avenue for further research.

Our results provide insight on the impact of temperature on protein synthesis and processing in *E. huxleyi*. Evidence has been provided to support the view that eukaryotic phytoplankton display higher rates of protein synthesis with increasing temperature, however this appears to be associated with increasing levels of protein misfolding and stress. At elevated temperatures, likely past the thermal optima for growth, it appears that cells experience considerable heat stress, inducing a relative up-regulation of repair proteins. The increase in protein synthesis observed with increasing temperature, and increased demand for repair proteins such as Hsps, likely incurs a considerable energetic cost and cellular demand for nutrients which may limit growth (O’Donnell et al., 2018). It is likely that the cost of repair played at least some part in driving lower growth rates observed in cultures acclimated to elevated- compared to moderate- warming treatments.

## 4. Summary and environmental implications

**Figure 5:**
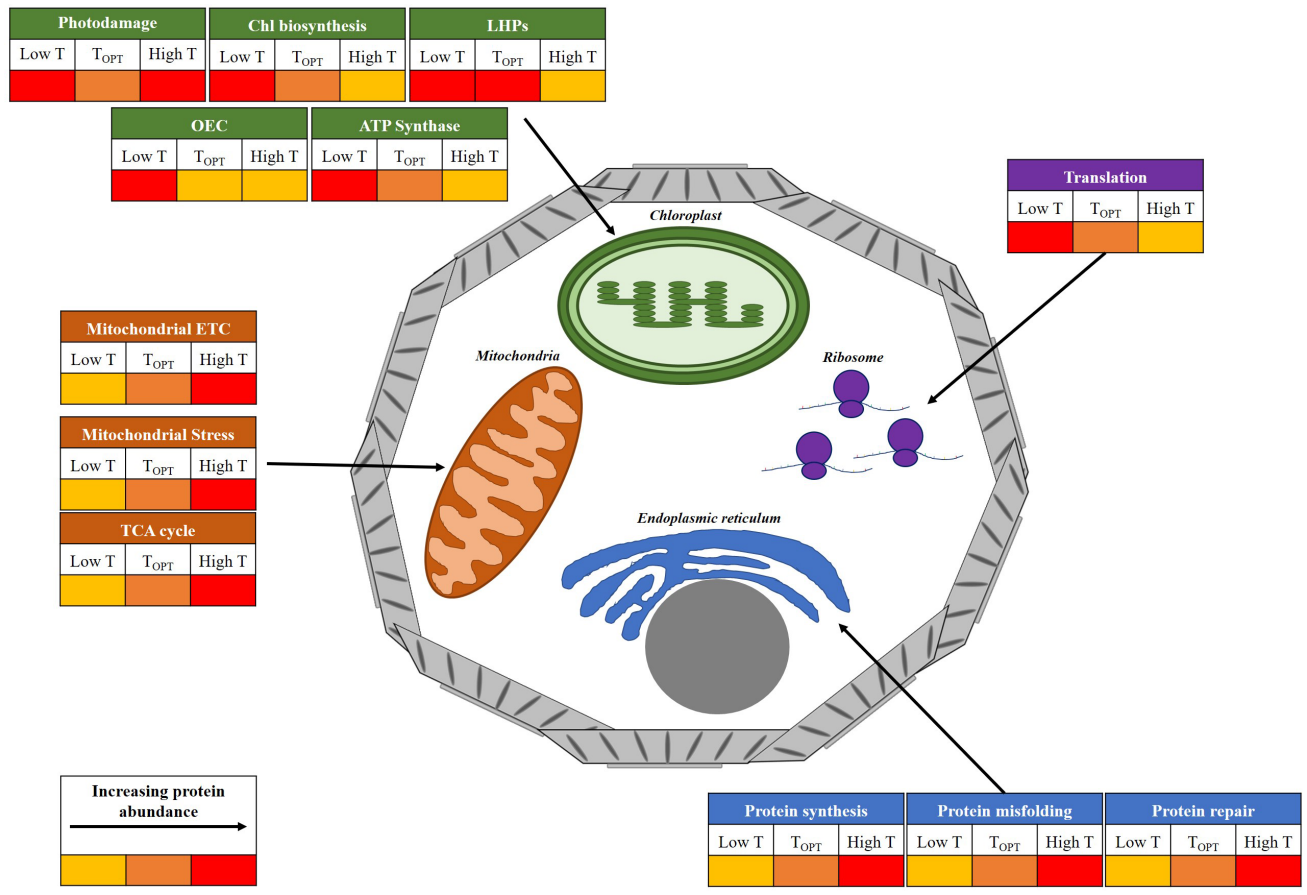
Illustrative diagram of the key cellular components and processes affected by temperature in *E. huxleyi*. The figure displays general changes in protein abundance at temperatures above (High T) and below (Low T) the thermal optima for growth (T_OPT_), inferred from proteomic analysis. Relative protein abundance at each temperature is represented by a colorimetric scale. Chlorophyll (Chl); Light harvesting proteins (LHPs); Oxygen-evolving complex (OEC); electron transport chain (ETC).

Increasing temperatures are the most direct impact of global climate change upon the ocean. In this study, using the ubiquitous coccolithophore *E. huxleyi*, we present proteomic insight to advance understanding of the molecular mechanisms that drive changes in phytoplankton cellular function as a result of warming. Our analysis reveals a modification of cellular function (Figure 5), which is largely in-line with existing literature on the altering performance of photosynthesis and respiration with increasing temperature. Critically, our work has been able to pull apart the mechanistic differences between acclimation to warming regimes representative of conditions above (elevated) and below (moderate) the optima for growth for this species. Additional measures of physiology (i.e., photosynthetic efficiency, net photosynthesis and respiration rates), coupled with focused investigation on the pathways highlighted in this study would greatly advance our understanding of these processes. Ultimately, the balance between photosynthetic and respiratory activity of marine phytoplankton alters the efficiency of the biological carbon pump in the ocean, hence temperature-induced changes in these processes will likely have knock-on effects on oceanic carbon cycling and the sequestration of carbon from the atmosphere (Barton et al., 2020). Additional research on taxa representing the key functional groups is required to confirm whether findings are consistent across marine phytoplankton, allowing for inference of the likely impact of warming on ocean biogeochemistry.

A temperature-dependent reduction in investment toward high-cost photosynthetic machinery was recorded in *E. huxleyi* RCC911, linked to the increase in efficiency of light harvesting and electron transport systems as temperatures rise, consistent with findings observed in land plants (Berry and Björkman, 1980; Havaux, 1993; Salvucci et al., 2001; Wise et al., 2004; Dutta et al., 2009; Efeoglu and Terzioglu, 2009). The down-regulation of chlorophyll biosynthesis observed under warming conditions appears to be a widely conserved feature of photosynthesis and may decrease the chlorophyll content of phytoplankton in the ocean, possibly compromising primary production measures based on satellite data (Lee et al., 2019; Cheng et al., 2022). Under elevated warming, believed past the thermal optima for growth, cultures appear to reduce the ratio of photosynthetic to respiratory ATP generation. Notably, our results show that even when experiencing some degree of heat stress *E. huxleyi* RCC911 is able to modulate its carbon metabolism to maximize growth by optimizing use of carbon metabolites, while by-passing the heat-sensitive complex I of the mitochondrial ETC. It remains to be investigated whether other *E. huxleyi* strains are able to modulate their metabolism in this fashion. Such research would greatly benefit our ability to evaluate the likely response of various oceanic zones to warming. Transcriptome analysis on *Phaeocystis globosa* indicated a remodeling of carbon fixation and up-regulation of TCA cycle activity, as photosynthesis-related transcripts were down-regulated in response to a combined warming and acidification treatment (Liang et al., 2023). This alongside increases in TCA cycle proteins observed in diatoms (Cheng et al., 2022), suggests some conservation of this metabolic response to warming across phytoplankton taxa. As a result of the metabolic alterations recorded, *E. huxleyi* RCC911 maintains a higher growth rate at high temperature (28°C) compared to cultures grown at lower temperature (17°C).

In terms of photosynthesis, our results suggest that warming in the sub-optimal to optimal thermal range may alleviate the costs associated with photosynthesis, due to its improving efficiency. Coupled with an increase in efficiency of translation, thus decreasing ribosomal investment, this will likely benefit growth. Hence, we can expect that under this scenario photosynthetic carbon fixation will be enhanced due to increasing cell number and photosynthetic activity. Simultaneously, rates of respiration are reported to rise with increasing temperature, typically at a greater rate than photosynthesis. As previously described, this causes a reduction in carbon-use-efficiency, thus decreasing the surplus energy available for growth and reducing incorporation of inorganic carbon into biomass. Past the photosynthetic thermal optima, this imbalance becomes more extreme, likely reducing carbon fixation. Importantly, our results highlight that the cost of repair appears a crucial limit to growth at elevated temperature, and likely constrains growth at extreme temperatures. In particular, it appears that under elevated warming the mitochondria experience a relatively high level of thermal stress. The elevated warming treatment examined in this study (+11°C relative to the control), is more relatable to a heat wave event. The response examined could be considered as an acclimated response, therefore it remains to be seen whether the same behavior would be observed during an acute response (i.e., hours to days). As the frequency of oceanic heat waves is becoming more frequent (IPCC, 2013; Oliver et al., 2021), it is important that we gather a clearer perspective of their likely impact on phytoplankton physiology.

Ultimately, the balance between photosynthesis and respiration, and the increasing cost of repair associated with rising ocean temperature will likely represent a species-specific adaptable trait, and will determine net carbon fixation by phytoplankton in the ocean. Research has shown species and inter-species variation in the thermal tolerance of marine phytoplankton (Thomas et al., 2012), as well as varying impact of temperature on photosynthesis and respiration of various phytoplankton taxa. The evolutionary history of individual species driven largely by their local environment will likely play an influential role in determining the threshold temperatures for photosynthesis, respiration and repair. Previous work has shown that rapid repair rather than enhanced thermal stability of enzymes facilitates growth at high temperatures in species adapted to high-temperature (O’Donnell et al., 2018). Such repair mechanisms are likely to vary substantially in species adapted to cooler temperatures, hence the cost of repair may represent a larger fraction of cellular carbon use in these species, or repair mechanisms may exhibit a lower threshold temperature before they are overwhelmed. However, conversely, polar and temperate phytoplankton species have been reported to display thermal optima considerably higher than the mean average temperature of their origin, whereas the thermal optima for tropical strains was closer or below their mean temperature (Thomas et al., 2012). As a result, it is predicted that species will display a poleward shift in their thermal niche as temperatures rise (Thomas et al., 2012), likely benefitting photosynthetic carbon fixation in lower latitudes if species move closer to their thermal optima. This is reported to not be the case in coccolithophores occupying low latitudes, where increases in temperature are predicted to considerably reduce growth, with potential impacts on carbon cycling and seawater alkalinity (Anderson et al., 2021). Above the thermal optima, carbon fixation becomes costly, thus in the absence of an acclimation, or indeed adaptation response, carbon fixation may be reduced due to increased costs of repair combined with reduced photosynthetic efficiency. Furthermore, during a heatwave event, where a species’ habitat is already frequently in the upper range of their tolerance, such metabolic adjustments might not be possible to sustain survival (Baker and Geider, 2021). Identifying the thermal optima and thresholds for each of photosynthesis, respiration and repair in phytoplankton occupying various oceanic zones, and indeed their propensity to adapt, will be critical to fully understand the implications of ocean warming and to inform future models on ocean biogeochemistry.

Much of the evidence available, including this study, examine the response of phytoplankton to temperature under nutrient replete conditions. The growth rates recorded and physiological alterations observed are likely to be much different under nutrient limitation (Thomas et al., 2017; Bestion et al., 2018; Aranguren-Gassis et al., 2019; Litchman and Thomas, 2023). Generally, increased temperature drives an increase in metabolic rate and hence higher demand for nutrients (Rhee and Gotham, 1981). A clear example of this can be seen in our work as the rates of protein synthesis and repair appear increased with increasing temperature. This increase in protein production drives an increased demand for major nutrients, as well as cofactors such as trace metals given that >50% proteins rely on metals for their function (Waldron et al., 2009). In particular, Fe is critical to the redox reactions of photosynthesis and respiration, and is believed to limit phytoplankton growth in 30%–40% of the global ocean (Sunda, 2012). Hence, it is likely that in areas where nutrients such as Fe are limited, species’ thermal optima will be reduced. Indeed, increased Fe availability is recorded to enhance thermal tolerance (Andrew et al., 2019). Alterations to metabolic processes under warming will likely alter the stoichiometry of marine phytoplankton, commonly examined in terms of their C:N:P content (Matsumoto et al., 2020). A fundamental concept behind this is the Redfield ratio (C_106_N_16_P), largely phytoplankton stoichiometry reflects the ratio of availability of nutrients in their natural environment (Redfield, 1934). The Redfield ratio has been found to vary between species and specific environmental conditions, including with temperature (Geider and La Roche, 2002; Spilling et al., 2015). In *E. huxleyi* the elemental composition of cells has been reported to alter under ocean warming, reported as increases in ratios of particulate inorganic carbon to particulate organic carbon, and polyunsaturated fatty acid content (Bi et al., 2018). The reduction of ribosomal content due to increasing efficiencies of protein synthesis at increased temperature is reported to increase the demand for N while reducing P requirement, thus increasing the N:P ratios of marine phytoplankton and subsequently may drive N-limitation (Toseland et al., 2013). It is possible that in our study, cultures exposed to elevated warming experienced a relative enhancement of N demand, due to the increasing costs of repair associated with heat stress in this treatment. The down-regulation of photosynthetic machinery in this treatment is in-line with previous reports of N-limitation in *E. huxleyi* and is consistent with that observed in land plants, suggesting that this regulatory process is well-conserved (Willows, 2006; Rokitta et al., 2014). However, evidence suggests that the supply of nutrients has a greater impact upon phytoplankton growth than temperature (Maranon et al., 2014). Additional studies are required to assess whether the alterations to core metabolic processes observed in phytoplankton under warming are consistent under nutrient limitation. Here, competing resource demands may result in a change in the thermal tolerance of photosynthesis and respiration, and hence alter carbon-use-efficiency (Gifford, 2003; Allison et al., 2010; Padfield et al., 2016; Barton et al., 2020). Ultimately, such knowledge is essential to predict the phytoplankton response to warming across the global ocean.

Of course, in the natural environment changes in temperature are more gradual, albeit happening at unprecedented rates. Therefore, phytoplankton will have the opportunity to adapt. Laboratory-based evolution studies have been utilized to examine the adaptive response to phytoplankton to increasing temperature. It has been reported that phytoplankton adapt to increasing temperature by reducing respiration rates, thus enhancing the efficiency at which photosynthetic carbon is allocated to growth (Padfield et al., 2016; Barton et al., 2020). Adaptation in this manner will likely mitigate any damages to the oceanic carbon sink caused by warming. Future work outlining the molecular mechanisms which drive such adaptation is of upmost importance. It is critical that we also consider that the marine phytoplankton make up a phototrophic community which will likely alter in composition as ocean temperatures rise, particularly in tropical regions (Thomas et al., 2012). Research is required to assess community-wide alterations in photosynthesis and respiration, to understand whether the cellular alterations identified in our study are consistent across photosynthetic species, or are specific to *E. huxleyi*. Clarification in this area will undoubtedly improve our ability to predict the overall impact of ocean warming on the contribution of marine phytoplankton to atmospheric carbon sequestration and input into the oceanic carbon sink.

## Data availability statement

The datasets presented in this study can be found in online repositories. The names of the repository/repositories and accession number(s) can be found in the article/Supplementary material.

## Author contributions

CD: conceptualization, investigation, formal analysis, writing—original draft, writing—review and editing, and visualization. SB: conceptualization and writing—review and editing. MF: methodology, resources, investigation, and writing—review and editing. RR: supervision, funding acquisition, conceptualization, and writing—review and editing. All authors contributed to the article and approved the submitted version.

## Funding

This work was funded by the European Research Council (ERC Consolidator Grant APPELS: ERC-2015-COG-681746 and ERC Advanced Grant SCOOBi: ERC-2020-ADG-101019146). Proteomic analysis was conducted at the Advanced Proteomics Facility, University of Oxford.

## Conflict of interest

The authors declare that the research was conducted in the absence of any commercial or financial relationships that could be construed as a potential conflict of interest.

## Publisher’s note

All claims expressed in this article are solely those of the authors and do not necessarily represent those of their affiliated organizations, or those of the publisher, the editors and the reviewers. Any product that may be evaluated in this article, or claim that may be made by its manufacturer, is not guaranteed or endorsed by the publisher.
